# Does Iterative Reconstruction Lower CT Radiation Dose: Evaluation of 15,000 Examinations

**DOI:** 10.1371/journal.pone.0081141

**Published:** 2013-11-26

**Authors:** Peter B. Noël, Bernhard Renger, Martin Fiebich, Daniela Münzel, Alexander A. Fingerle, Ernst J. Rummeny, Martin Dobritz

**Affiliations:** 1 Department of Diagnostic an Interventional Radiology, Technische Universität München, Munich, Germany; 2 Institute of Medical Physics and Radiation Protection, University of Applied Sciences, Giessen, Germany; The University of Chicago, United States of America

## Abstract

**Purpose:**

Evaluation of 15,000 computed tomography (CT) examinations to investigate if iterative reconstruction (IR) reduces sustainably radiation exposure.

**Method and Materials:**

Information from 15,000 CT examinations was collected, including all aspects of the exams such as scan parameter, patient information, and reconstruction instructions. The examinations were acquired between January 2010 and December 2012, while after 15 months a first generation IR algorithm was installed. To collect the necessary information from PACS, RIS, MPPS and structured reports a Dose Monitoring System was developed. To harvest all possible information an optical character recognition system was integrated, for example to collect information from the screenshot CT-dose report. The tool transfers all data to a database for further processing such as the calculation of effective dose and organ doses. To evaluate if IR provides a sustainable dose reduction, the effective dose values were statistically analyzed with respect to protocol type, diagnostic indication, and patient population.

**Results:**

IR has the potential to reduce radiation dose significantly. Before clinical introduction of IR the average effective dose was 10.1±7.8mSv and with IR 8.9±7.1mSv (p*=0.01). Especially in CTA, with the possibility to use kV reduction protocols, such as in aortic CTAs (before IR: average14.2±7.8mSv; median11.4mSv /with IR:average9.9±7.4mSv; median7.4mSv), or pulmonary CTAs (before IR: average9.7±6.2mSV; median7.7mSv /with IR: average6.4±4.7mSv; median4.8mSv) the dose reduction effect is significant(p*=0.01). On the contrary for unenhanced low-dose scans of the cranial (for example sinuses) the reduction is not significant (before IR:average6.6±5.8mSv; median3.9mSv/with IR:average6.0±3.1mSV; median3.2mSv).

**Conclusion:**

The dose aspect remains a priority in CT research. Iterative reconstruction algorithms reduce sustainably and significantly radiation dose in the clinical routine. Our results illustrate that not only in studies with a limited number of patients but also in the clinical routine, IRs provide long-term dose saving.

## Introduction

During the last years, Iterative Reconstruction (IR) algorithms for diagnostic Computed Tomography (CT) have found great popularity in the clinical community due to their dose reduction potentials. Each of the CT vendors has introduced their own flavor of such reconstruction methods [1-6]. For each of these methods it is claimed that the image noise can be significantly reduced, which, in turn, consequently allows for reduction of radiation exposure. Some techniques claim to consider the individual noise in each detector channels; others, which are more advanced, claim superiority by additionally modeling the system geometry and the system physics more accurately. Independent of the availability of the algorithmic details of those methods, the promised dose reductions from IRs are of high interest, since CT remains by far the most important source of medical radiation exposure [7].

Several investigators have presented phantom as well as clinical studies for evaluation of different IR algorithms with respect of dose reduction, image quality, and diagnostic confidence [8-16]. Especially in clinical evaluations, with number of patients enrolled in a study ranging from ten up to several 100, it has been shown that dose reductions depending on the clinical indication and protocol are achievable [8-16]. The image quality / impression of the newly discovered techniques did not directly receive highest acceptance by clinicians. Even with the illustrated possible dose reductions, this poses the risk that over time, during the clinical routine, the protocols are transformed back to the previous image impression and thus to higher dose levels. 

In this study we monitored the effective dose of 15,000 CT examinations before and after introduction of a first generation IR algorithm. To evaluate if IR provides a sustainable dose reduction, the effective dose values were statistically analyzed with respect to protocol type, diagnostic indication, and patient population.

## Materials and Methods

### CT Acquisition

This single-center study was IRB approved. For each participant a written consent was obtained, as approved by the ethical committee at the Faculty of Medicine of the Technische Universität München. The study included 14854 patients (6971 before & 7883 after installation of the IR system) who underwent a CT examination between January 2010 and December 2012. All patients were imaged on the same 256-slice CT scanner (Brilliance iCT, Philips Healthcare, Cleveland, OH, United States). The departmental CT protocols were employed; this procedure involves for some protocols a Body-Mass-Index (BMI) adjustment of tube-current and latest dose modulation techniques. After 15 months a first-generation IR algorithm was added to the system. As guidance for adjusting the initial imaging parameter, we used experiences we gained during a prototype evaluation with phantoms and retrospective collected patient data (for details please see 12). Consequently each protocol was adjusted over a period of time with respect to image parameters to achieve the minimal dose while maintaining diagnostic quality. The diagnostic quality is defined as a combination of objective and subjective image quality metrics. We evaluated all metrics in regular meetings to control progress of the parameter adjustment. Note, the evaluation and optimization of the diagnostic quality are described in detail in the discussion of this manuscript. 

### Dose Monitoring System (DMS)

The DMS tool was developed to collect examination, patient, and modality information. For data collection, we integrated data extracted from a HIS/RIS system (SAP, IS-Hmed*, CSC, Falls Church, VA, United States), a direct access to a PACS-Database (EasyAccess, Philips Healthcare, Best, The Netherlands), MPPS-data, and Dicom-SR. The integration of all sources overcomes the weaknesses of single systems, for example incorrect user input into the RIS system. These corrupted data can be identified and corrected by using information from multiple sources. Further, some crucial information is only presented in DICOM-images or DICOM-overlays. To overcome this obstacle we integrated an optical character recognition system to extract, for example, information from CT-dose reports. All collected data are transferred to a database (MySQL, Oracle, Redwood Shores, CA, United States) for presentation and further evaluation, e.g., for calculations of effective as well as organ doses. Since the implementation of your PACS-System in 2004, HIS/RIS-data and PACS-data are available in the database.

### Data Analysis

For data-analysis and calculation of dose values a data-analyzing software (QlikView, Qliktech, Radnor, PA, United States) was used. For estimation of effective dose values the dose length product (DLP), calculated from the CTDI_vol_ times the scan length, is multiplied by an anatomical-region-specific conversation factor [17,18]. The conversation factors for different anatomical regions are system-specifically corrected depending on the DLP_air_, the scanner geometry as well as the different tube voltages [19]. Standard CT systems report the DLP based on a 16 cm or 32 cm reference phantom; however, the correction with regard to system specifics like DLP_air_ and the scanner geometry improve the calculation of effective dose values up to 20% (for more details see 19,20). For quality insurance one hundred random samples of each protocol type were compared to results from the dose estimation software CT-Expo V1.7.1 [20]. The evaluation showed a maximum error of ±5.0% for the final effective dose values. The error was caused by a superior definition of the different organ regions when using CT-Expo. Note, for the future we are planning to update our system to better define different organ regions. To evaluate if IR provides a sustainable dose reduction, the effective dose values were analyzed with respect to protocol type, diagnostic indication, frequency of examination, and patient population. To provide an easier access to the data, the acquisitions are combined into quarter years. Data for each quarter year are expressed as median and arithmetic mean ± SD. A two-tailed paired Student t-test was performed for comparison of effective dose levels of different time periods. A p-value ≤ 0.01 was considered to indicate statistical significance. All statistics were computed with Microsoft Excel.

## Results

**Figure 1:**
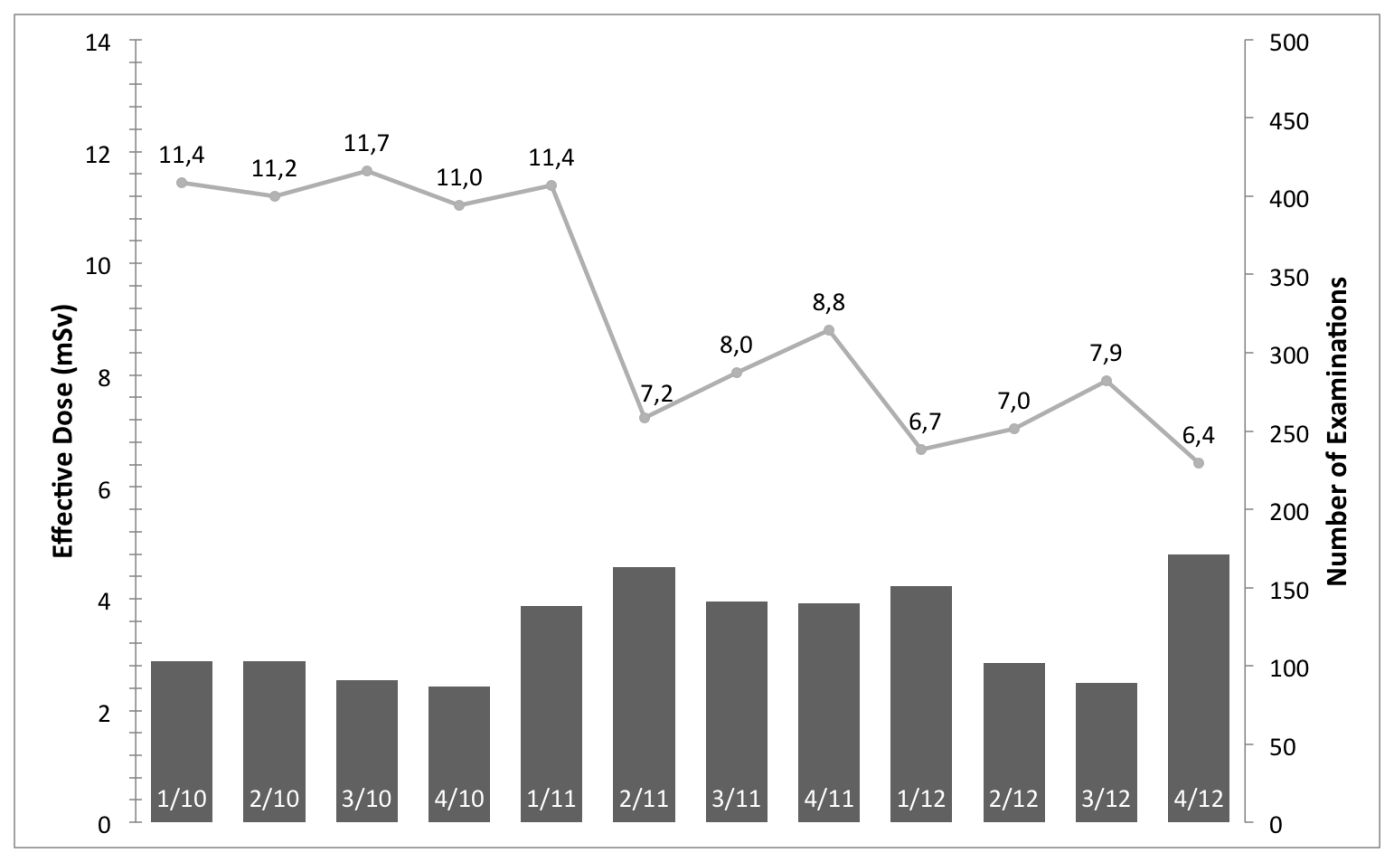
The median effective dose and number of examinations per quarter year in mSv for aortic CTA examinations. With the introduction the IR system (between 1/11 and 2/11) a significant reduction in radiation exposure can be reported.

**Figure 2:**
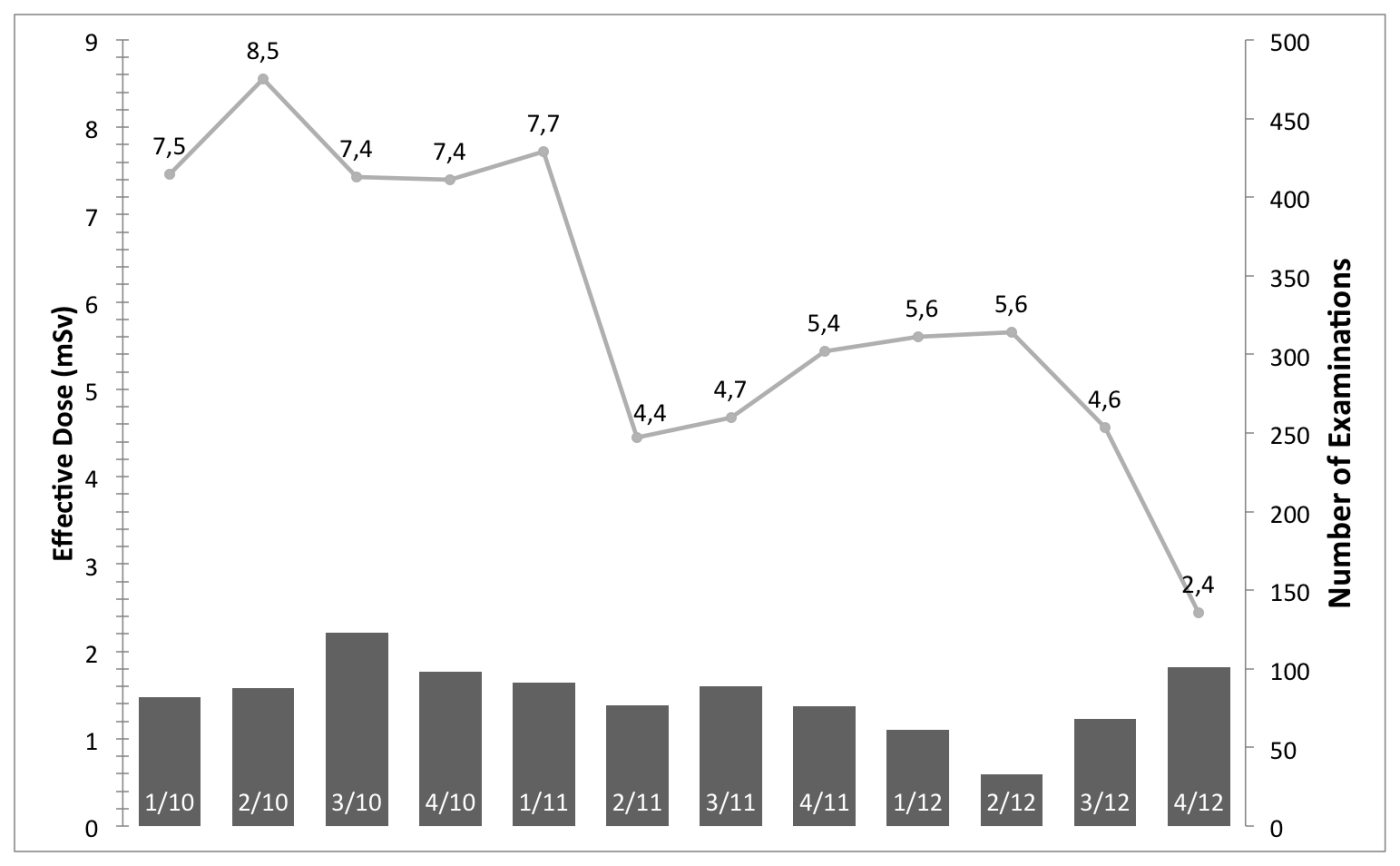
The median effective dose and number of examinations per quarter year in mSv for pulmonary CTA examinations. The effective dose drops significantly in the last reported quarter, since the protocol was switched from 120kv to 100kv.

**Figure 3:**
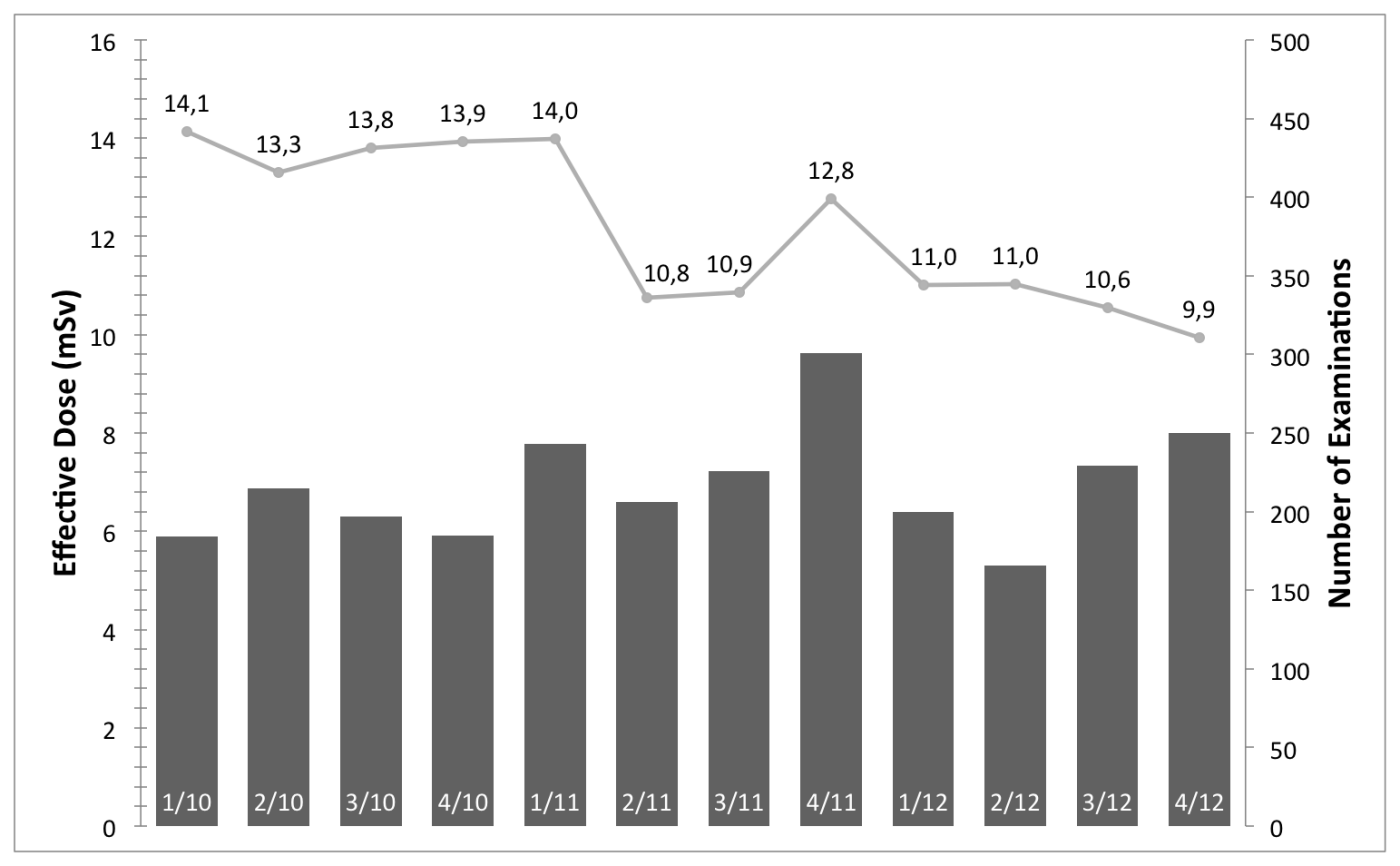
The median effective dose and number of examinations per quarter year in mSv for thorax-abdomen-pelvis CT examinations. With the introduction the IR system (between 1/11 and 2/11) a significant reduction in radiation exposure can be reported. The clinical value of a DMS is demonstrated with the increase in effective dose in 4/11 and the direct detection and correction.

**Figure 4:**
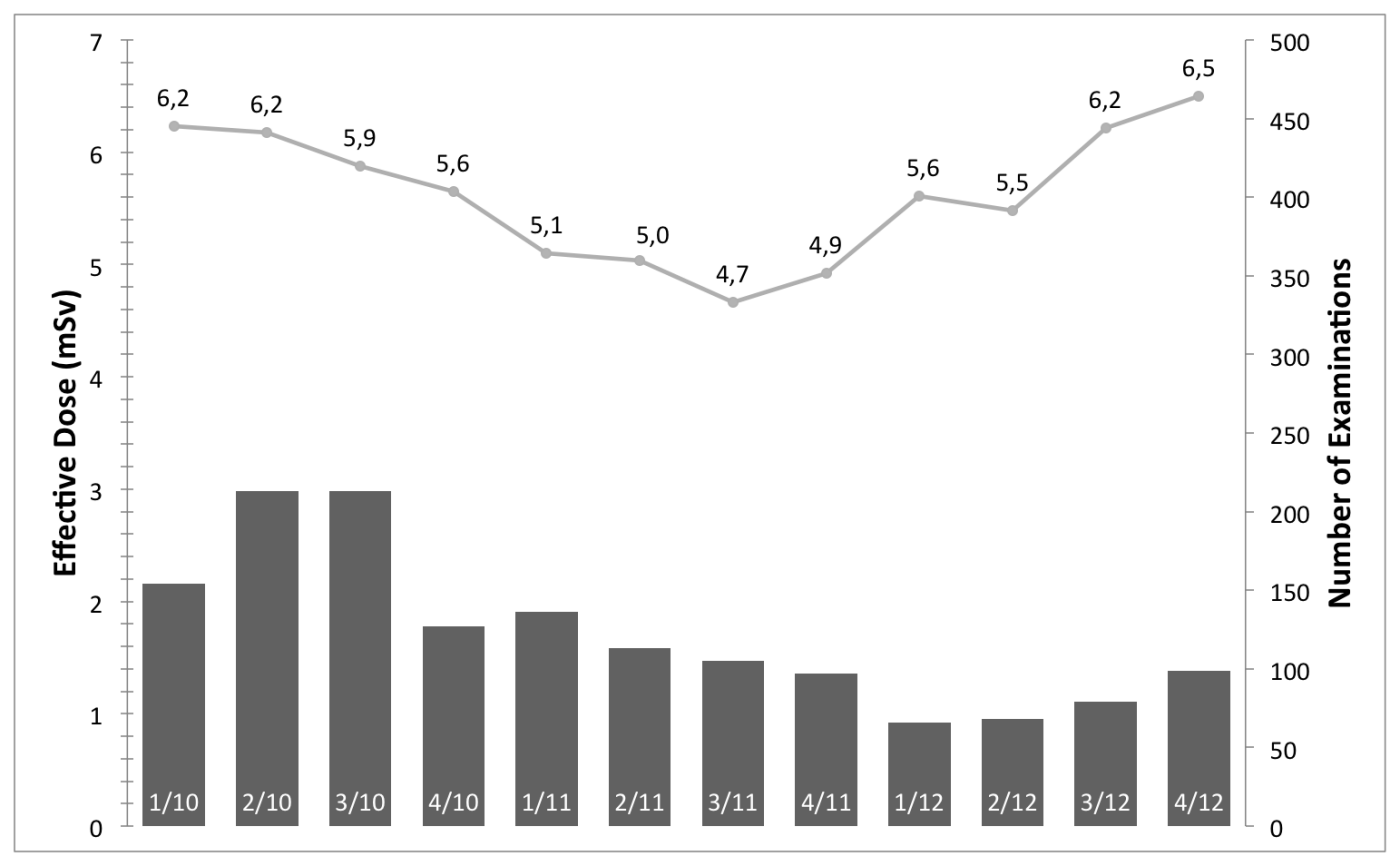
The median effective dose and number of examinations per quarter year in mSv for low dose scans of the cranial (for example sinuses). Note it is important to point out that for unenhanced neurological CT scan the effect of IR in combination with a wide-detector configuration is naturally minor.

Figure 1 shows the median effective dose and examinations per quarter year in millisievert (mSv) for aortic CTA examinations. With respect to the effect of IRs before and after introduction on the radiation exposure, an average of 14.2 ± 7.8 mSv and median of 11.4 mSv, and average of 9.9 ± 7.4 mSv and median of 7.4 mSv are reported, respectively. Figure 2, 3, & 4 have an analog layout as Figure 1, while the examination types are pulmonary CTA, thorax-abdomen-pelvis CT, and unenhanced cranial CT scans. For the pulmonary CTAs (see Figure 2) the effective dose dropped from average of 9.7 ± 6.2 mSV and median of 7.7 mSv to average of 6.4 ± 4.7 mSv and median of 4.8 mSv. Note for Figure 2, the effective dose drops significantly in the last reported quarter, since the protocol was switched from 120 kv to 100 kv. This has been reported previously that IR allows a higher reduction in radiation exposure when it comes to high-contrast acquisitions. The IR can compensate the additional noise caused by the reduction of tube-voltage [9,21]. For the thorax-abdomen-pelvis CTs (see Figure 3), mostly used for cancer staging, the effective dose dropped from average of 14.6 ± 5.0 mSv and median of 13.8 mSv to average of 12.5 ± 5.5 mSv and median of 11.0 mSv. For CTA, pulmonary CTA, and thorax-abdomen-pelvis CT examinations the dose reduction after introduction of the IR system was statistically significant (p*=0.01). For unenhanced low-dose scans of the cranial (for example sinuses) the effective dose reduction is not significant as our statistical analysis revealed. The average and median effective dose was 6.6 ± 5.8 mSv and 3.9 mSv with conventional Filtered Backprojection 6.0 ± 3.1 mSV and a median of 3.2 mSv with IR (see Figure 4). It is important to point out that for unenhanced neurological CT scan the effect of IR in combination with a wide-detector configuration is naturally minor. Other investigator confirmed this observation in studies with one hundred examinations [22]. 

**Figure 5:**
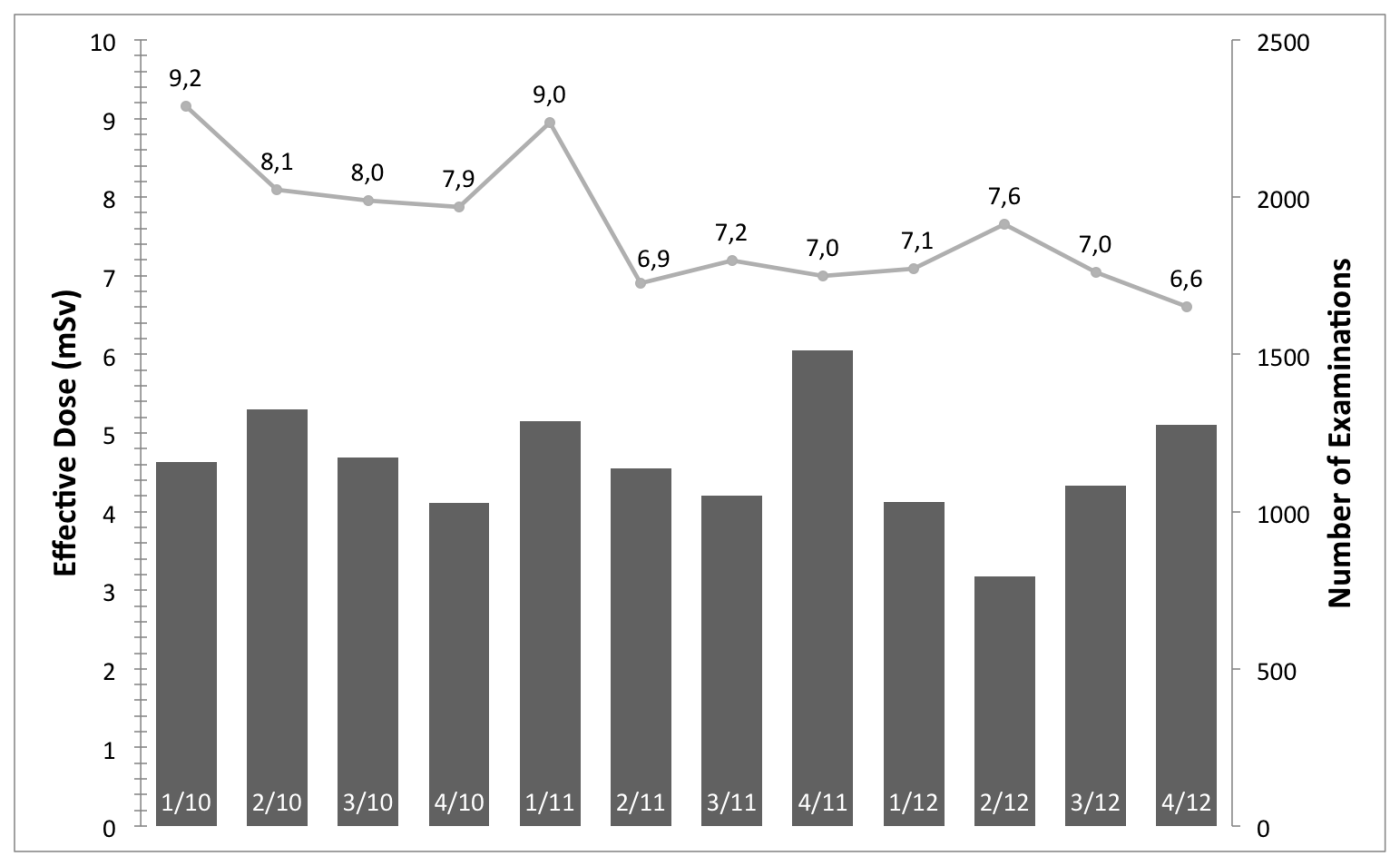
The median effective dose and number of examinations per quarter year in mSv for all examination between January 2010 and December 2012. With the introduction the IR system (between 1/11 and 2/11) a significant reduction in radiation exposure can be reported.

Figure 5 illustrates that IR has an impact to reduce radiation dose significantly (p*=0.01). Before clinical introduction of IR the average effective dose over all scans was 10.1 ± 7.8 mSv and with IR 8.9 ± 7.1 mSv. Note, the cumulative dose values between different radiology departments are strongly dependent on the type of patient population; thus, between different departments the cumulative dose can be significantly different. Table 1 & 2 summarizes all presented examination types and the collected dose information of all examinations. 

**Table 1:** Overview of the collected data for different examination types (# number of examination, μ average effective dose, χ median effective dose, σ standard deviation of effective dose).

**Quartal**	**#**	**μ (mSv)**	**χ (mSv)**	**σ (mSv)**	**Quartal**	**#**	**μ (mSv)**	**χ (mSv)**	**σ (mSv)**
**CT Angiography (Abdomen)**					**CT Thorax-Abdomen-Pelvis**				
1/10	103	13,19	11,44	5,89	1/10	184	15,25	14,12	3,95
2/10	103	14,48	11,21	10,78	2/10	215	12,91	13,31	5,35
3/10	91	14,80	11,65	7,64	3/10	197	14,75	13,80	5,39
4/10	87	13,72	11,04	7,46	4/10	185	15,11	13,93	5,40
1/11	138	13,54	11,40	7,11	1/11	243	14,95	13,99	4,84
2/11	163	9,56	7,24	5,71	2/11	206	11,92	10,75	4,65
3/11	141	10,09	8,04	6,67	3/11	226	12,20	10,86	5,17
4/11	140	11,58	8,81	8,43	4/11	301	14,84	12,77	6,95
1/12	151	9,21	6,66	9,15	1/12	200	12,57	11,02	5,23
2/12	102	9,65	7,04	7,30	2/12	166	12,56	11,04	5,59
3/12	89	10,65	7,90	8,31	3/12	229	12,22	10,55	5,92
4/12	171	8,29	6,43	6,54	4/12	250	11,23	9,93	4,97
**CT Angiography (Embolie)**					**CT Cranium (nativ)**				
1/10	82	9,81	7,46	6,24	1/10	154	7,12	6,23	4,80
2/10	88	9,74	8,55	6,41	2/10	213	6,91	6,17	3,95
3/10	123	10,29	7,43	6,82	3/10	213	6,86	5,87	3,58
4/10	98	9,09	7,40	5,50	4/10	127	6,16	5,65	3,41
1/11	91	9,54	7,72	5,92	1/11	136	6,02	5,10	3,99
2/11	77	5,41	4,44	3,16	2/11	113	5,55	5,04	2,61
3/11	89	5,94	4,67	4,07	3/11	105	5,19	4,66	1,94
4/11	76	6,38	5,43	3,34	4/11	97	5,52	4,92	2,69
1/12	61	8,72	5,60	8,79	1/12	66	6,84	5,61	6,09
2/12	33	8,01	5,65	5,65	2/12	68	5,88	5,48	2,12
3/12	68	6,09	4,56	3,89	3/12	79	7,13	6,21	5,04
4/12	101	4,20	2,44	3,92	4/12	99	6,94	6,50	3,87

**Table 2:** Overview of the collected data for all examination between January 2010 and December 2012 (# number of examination, μ average effective dose, χ median effective dose, σ standard deviation of effective dose).

**Quartal**	**#**	**μ (mSv)**	**χ (mSv)**	**σ (mSv)**
1/10	1158	10,35	9,15	7,58
2/10	1324	9,98	8,09	7,87
3/10	1173	9,98	7,95	7,53
4/10	1029	9,99	7,87	7,13
1/11	1287	10,33	8,95	7,18
2/11	1137	8,41	6,91	6,28
3/11	1050	8,74	7,20	6,92
4/11	1513	10,01	6,99	8,37
1/12	1030	8,84	7,09	7,56
2/12	795	9,17	7,65	6,72
3/12	1082	8,62	7,04	7,51
4/12	1276	7,79	6,61	6,37

*Total of 14854 examinations were enrolled into this study* (6971 before & 7883 after installation of the IR system).

## Discussion

In this study we observed that when employing an IR algorithm in the clinical day-to-day routine, it is possible to sustainably and significantly reduce radiation exposure in diagnostic MDCT examinations. The amount of radiation dose reduction cannot be generalized to a fixed amount since the initial dose and the reduced dose depend on several parameters, which include diagnostic indication, protocol design, and even personal bias towards the one or the other image quality. The last point is one of the main challenges in the current and future integration of IRs into the clinical routine. Regularly the image quality / impression of the newly introduced reconstruction techniques do not directly receive highest acceptance by clinicians; thus, a longer time period is necessary to adjust parameters to achieve a perfect balance. For example, in our case, each protocol was adjusted over a period of time with respect to image parameters to achieve the minimal dose while maintaining the diagnostic quality. Diagnostic quality is a combination of image quality and the indication of a given examination. Image quality can be measured with several different metrics, which include contrast-to-noise ratio, image noise, resolution and many more. Important is that one recognizes that theses metrics alone do not prove the diagnostic merit of a reconstruction. For example, one could use a strong image filter to totally eliminate image noise while as a drawback such a filter would increasing blurring and would subsequently strongly limit the diagnostic quality. To determine if the reconstruction has diagnostic merit for a specific indication, one uses subjective image assessment. Thus the indication of the examination, in combination with all these metrics (objective and subjective), plays a mayor role to evaluate the diagnostic quality. In our case, if not more frequently demanded, a weekly meeting was held to review the current diagnostic quality. The meetings included three radiologists (several years of experience in CT diagnostics) and two medical physicists. Main focus was to keep the radiation exposure minimal while ensuring optimal diagnostic quality (as legally mandatory in Germany). This optimization process can take several months because, first of all, the process should happen in a gradual process to ensure diagnostic quality and secondly the adjustment to the new image impression takes some time. On this note, in our case we optimized our protocols initially when the system was installed (conventional FBP reconstruction was used). Thus when the IR system was installed all protocols were re-optimized. As guidance for adjusting the initial imaging parameter, we used experiences we gained during a IR prototype evaluation with phantoms and retrospective collected patient data (for details please see 12). Further details on how to perform such an optimization process can be found in [23].

The topic of IR algorithms for CT application had a strong revival in the last years, considering that the number of publications focusing on this topic has tripled in the last five years [24]. This popularity can be lead back to the general interest to reduce radiation dose in combination with a higher availability of parallel computing tools. On this note, IR is not the only possibility to reduce radiation exposure; other current and future options can be found in [25]. With regard to most publications, the evaluation of IR algorithms was done on a limited number of patient cases. In our case we could consider a large number of patient cases to evaluate long-term effects. However, compared to the work of others, we could not consider the diagnostic quality for all cases, but reviewed the current diagnostic quality as well as the radiation exposures in a weekly meeting. 

## Conclusion

The dose aspect in diagnostic CT remains one of the highest priorities. Over the last decade several steps have been implemented toward dose reduction, which indicates that we are moving toward effective dose levels of less than 1 mSv. Important for the clinical day-to-day routine is to realize that if a CT scan is justified from a medical point of view, then the dose should be secondary while all dose reduction options should be fully utilized to a level where diagnostic confidence is ensured. In conclusion, our results illustrate that not only in studies with a limited number of patients but also in the clinical routine, IRs provide long-term dose savings.
